# Complement as a vital nexus of the pathobiological connectome for acute respiratory distress syndrome: An emerging therapeutic target

**DOI:** 10.3389/fimmu.2023.1100461

**Published:** 2023-03-17

**Authors:** Zhangsheng Yang, Susannah E. Nicholson, Tomas S. Cancio, Leopoldo C. Cancio, Yansong Li

**Affiliations:** ^1^ Combat Casualty Care Research Team (CRT) 3, United States (US) Army Institute of Surgical Research, Joint Base San Antonio (JBSA)- Fort Sam Houston, TX, United States; ^2^ Division of Trauma Research, University of Texas Health Science Center at San Antonio, San Antonio, TX, United States; ^3^ United States (US) Army Burn Center, United States (US) Army Institute of Surgical Research, Joint Base San Antonio (JBSA)- Fort Sam Houston, TX, United States; ^4^ The Geneva Foundation, Immunological Damage Control Resuscitation Program, Tacoma, WA, United States

**Keywords:** ARDS, complementome, multiome, connectome, pathobiology, therapeutics, clinical trial, COVID-19

## Abstract

The hallmark of acute respiratory distress syndrome (ARDS) pathobiology is unchecked inflammation-driven diffuse alveolar damage and alveolar-capillary barrier dysfunction. Currently, therapeutic interventions for ARDS remain largely limited to pulmonary-supportive strategies, and there is an unmet demand for pharmacologic therapies targeting the underlying pathology of ARDS in patients suffering from the illness. The complement cascade (ComC) plays an integral role in the regulation of both innate and adaptive immune responses. ComC activation can prime an overzealous cytokine storm and tissue/organ damage. The ARDS and acute lung injury (ALI) have an established relationship with early maladaptive ComC activation. In this review, we have collected evidence from the current studies linking ALI/ARDS with ComC dysregulation, focusing on elucidating the new emerging roles of the extracellular (canonical) and intracellular (non-canonical or complosome), ComC (complementome) in ALI/ARDS pathobiology, and highlighting complementome as a vital nexus of the pathobiological connectome for ALI/ARDS *via* its crosstalking with other systems of the immunome, DAMPome, PAMPome, coagulome, metabolome, and microbiome. We have also discussed the diagnostic/therapeutic potential and future direction of ALI/ARDS care with the ultimate goal of better defining mechanistic subtypes (endotypes and theratypes) through new methodologies in order to facilitate a more precise and effective complement-targeted therapy for treating these comorbidities. This information leads to support for a therapeutic anti-inflammatory strategy by targeting the ComC, where the arsenal of clinical-stage complement-specific drugs is available, especially for patients with ALI/ARDS due to COVID-19.

## Highlights

• ARDS is a life-threatening syndrome without specific pharmacotherapy for ARDS thus far.
• The role of profoundly destructive influence of unchecked ComC-driven air-blood-barrier failure, SIRS, immunothrombosis, meta-inflammation, PICS, and dysbiosis has been extensively studied in preclinical and clinical ARDS/ALI.
• ComC activation products (C3a, C4d, C5a, C5b-9, Bb) and their corresponding receptors provide useful diagnostic tools and promising therapeutic targets.
• With extreme heterogeneity in ARDS, the future of ARDS management strives towards identification of endotypes and theratypes through new methodologies and delivery of precision therapeutic interventions.
• Prospective studies are needed to confirm evidence of endotype-/theratype-driven therapeutic response prior to incorporation into clinical practice.

## Open questions

• Is the composition of the complosome universal or cell specific?
• Which do the exact cellular subcompartments contain complement components, receptors and regulators for activation fragments?
• How does the complosome orchestrate the intracellular organelles (mitochondria, lysosomes, endoplasmic reticulum, etc.) and cytoskeleton?
• How does the complosome dance with the intracellular DAMPs and PAMPs?
• How does the complementome act in concert with other mutiomes to contribute to ARDS pathobiology?

## 1 Introduction

2022 marks the 55^th^ year anniversary of the first description of acute respiratory distress syndrome (ARDS). Since then, substantial progress has been made in identifying the risk factors for and the pathogenic contributors to ARDS with an emphasis on the mechanisms of injury to the pulmonary endothelium and the epithelium. Despite considerable progress of pathophysiological mechanisms in the last five decades, no effective pharmacological therapeutics are available for ARDS patients yet. Current treatments still largely rely on supportive approaches of lung protective ventilation and a conservative fluid resuscitation (1, 2). Mortality remains high at 40%, and for patients who survive, recovery continues for months or even years. Recent studies have shown that uncontrolled complement cascade (ComC) activation may contribute to the pathological processes of ARDS (3–6). Therefore, modulation of the ComC may hold great promise for the treatment of acute lung injury (ALI) and ARDS. This review provides a broad overview of the ComC role in ALI and ARDS, discusses current understanding of the comprehensive molecular mechanistic insights of complement activation-mediated-ALI/ARDS, and highlights the newest developments in ComC pharmacological interventions for ARDS to help understand the rationale behind the current novel development of treatment strategies targeting ComC in ALI/ARDS patients.

## 2 ARDS

ARDS is a form of non-cardiogenic pulmonary edema, due to alveolar injury secondary to an inflammatory process. ARDS is a life-threatening condition of critically-ill patients characterized by acute onset of the illness, bilateral lung infiltrates, and respiratory distress due to severe arterial hypoxemia (7). This disorder is associated with pulmonary endothelial injury and alveolar epithelial damage. It carries a high mortality rate (8) without any effective pharmacological interventions.

### 2.1 Epidemiology

It has been reported that approximately 200,000 cases occur in the United States annually (8), with poor clinical outcomes with a pooled mortality rate of approximately 40% (8). Twenty-five percent of ARDS cases are classified as mild and 75% as moderate or severe. However, a third of the mild cases can progress to moderate or severe disease (9).

### 2.2 Pathophysiology and etiology

**Figure 1:**
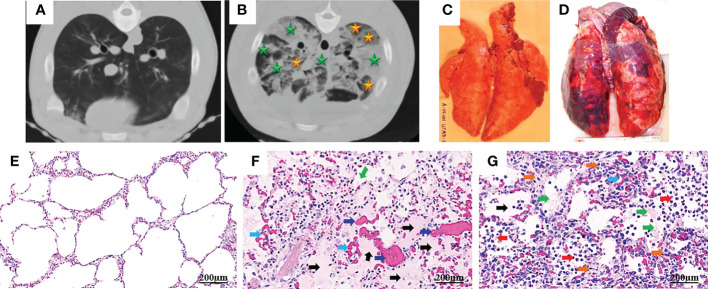
Computed tomography (CT) and histopathology of ARDS following smoke inhalation and burn in pigs. Anesthetized Yorkshire female pigs (35-50kg) were injured by a combination of wood bark smoke inhalation (the cooled smoke delivered with a dose of 20-30 liters at a tidal volume of 30 ml/kg) and a 40% total body surface area deep burn, and observed under a standard ARDSNet mechanical ventilation for 48 hours. The lung tissues were harvested at the end of study for macro-/micro-pathological evaluation and CT scan was conducted at the end of study. Chest CT from a non-injured lung **(A)** and an ARDS lung showing bilateral infiltration (**B**, golden star, ground glass opacities; green star, consolidation areas). Macropathology from a non-injured lung **(C)** and an ARDS lung **(D)**. Lung from a non-injured pig **(E)** and an ARDS pig **(F, G)**, stained with hematoxylin and eosin. The histological alterations of ARDS are characterized by alveolar (black arrow) and interstitial (golden arrow) edema, hyaline membrane formation (green arrows), inflammatory cell infiltration (red arrow), thrombosis (dark blue arrow), and vascular congestion (light blue arrow) (14, 15).

ARDS represents a stereotypic response to various etiologies. As shown in **Table 1**, its pathological changes have been described as three overlapping phases: exudative (acute), proliferative (sub-acute) and fibrotic (chronic) phases (11, 12). The hallmark of the acute phase is alveolar-capillary barrier dysfunction driven by immune-cell-mediated destruction of alveolar epithelium and vascular endothelium (11, 13), and is characterized by bilateral infiltration (14) (**Figures 1A, B**), interstitial and alveolar edema, capillary congestion, intra-alveolar hemorrhage, inflammatory cell infiltration, diffuse alveolar damage (DAD), intra-alveolar and intravascular fibrin deposition, hyaline membrane formation, and microthrombi (14, 15) (**Figures 1C-G**). These alterations lead to hypoxia, hyperinflammation, metabolic acidosis, pulmonary hypertension, and increased mortality. Traditionally, ARDS is recognized to be a neutrophil-driven disease. However, accumulating evidence has demonstrated the involvement of innate immune cells such as macrophages and platelets, and the adaptive immune system in the pathogenesis of ARDS. The proliferative phase occurs early (within the initial three days) and attempts to reduce the damage caused during the acute phase and restore pulmonary function with the regeneration of alveolar type II cells, differentiation of alveolar type II cells to alveolar type I cells, and the proliferation of fibroblasts and myofibroblasts. The fibrotic phase develops due to a failure of removal of alveolar collagen, which results in an increased dead-space fraction, high minute ventilation requirement, pulmonary hypertension, and further reduction of lung compliance. A hallmark of the damage seen in ARDS is that it is not uniform.

**Table 1:** Pathophysiology of ARDS (10).

Phases	Mechanisms	Pathophysiology	Clinical outcomes
Exudative phase (Acute phase)	Immune-cell-mediated destruction of pulmonary endothelium and epithelium that leads to increasing vascular permeability, decreasing epithelial water clearance, loss of surfactant production	• Alveolar Edema • Inflammatory cell infiltration • Intra-alveolar/intravascular fibrin deposition and hyaline membrane (HM) formation • Diffused alveolar damage • Consolidation and atelectasis • Poor oxygen diffusion • Intrapulmonary shunting	• Hypoxia • Hyperinflammation • Metabolic acidosis • Pulmonary hypertension • Higher vasopressor requirement • Increased mortality
Proliferative phase (Sub-acute phase)	Regenerative reparation	• Regeneration of alveolar type II cells • Alveolar type II cells differentiation to Alveolar type I cells • Proliferation of fibroblasts and myofibroblasts	• Attenuated hypoxia • Increased clearance of exudative fluid • Enhanced phagocytosis • Fibrosis
Fibrotic phase (Chronic phase)	Regenerative reparation (failure of removal of alveolar collagen, organizing fibrosis)	• Increased dead-space fraction • Reduction in lung compliance	• Hypoxia • High minute ventilation requirement • Pulmonary hypertension

ARDS has many risk factors with heterogeneous etiologies. Generally, ARDS is classified into two major types: direct lung injury affecting the pulmonary epithelium and indirect lung injury disrupting the pulmonary vascular endothelium. Greater understanding of the differences between direct and indirect lung injury may help development of new therapies targeted at specific subgroups of patients with ARDS. The common causes of ARDS are summarized in **Table 2**. There are certain common pathophysiological differences between direct and indirect lung injury (10) (**Table 2**).

**Table 2:** Features that may differ between direct and indirect ARDS (14, 16–18).

	Direct ARDS	Indirect ARDS
Common causes	• Pneumonia (bacterial, viral, fungal, Parasitic) • Aspiration • Mechanical ventilation (baro-/volu-trauma) • Lung contusion • Toxic inhalation • Near-drowning • Graft failure of lung transplantation • Reperfusion injury	• Sepsis • Non-thoracic trauma • Massive transfusion • Acute pancreatitis • Drug overdose • Burn injury • Traumatic brain injury (neurogenic) • Disseminated intravascular coagulation • Embolism (fat, amniotic)
Pathology	• Pronounced epithelial injury • More alveolar destruction • More severely impaired alveolar fluid clearance • More edema • Discontinuous and thick hyaline membrane • More neutrophil infiltration and apoptosis • Persistent inflammation • Regulatory T cell infiltration at late stage • IL-6 as more anti-inflammatory • Surfactant loss • Profound fibrin and collagen	• Pronounced endothelial injury • More interstitial edema • Less severely impaired alveolar fluid clearance • Less edema • Even and thin hyaline membrane • More monocyte infiltration • Quick inflammation resolution • Regulatory T cell infiltration at early stage • IL-6 as more pro-inflammatory • Normal surfactant • Predominant factor VIII
Physiology	• Persistent abnormalities • Increased lung elastance	• Rapidly resolved abnormalities • Increased lung and chest wall elastance
Radiography	• Consolidation equivalent to ground glass opacities • More diffuse ground glass opacities • More asymmetric and patchy consolidation • Opacities located in the non-dependent lung regions	• Ground glass opacities more prominent than consolidation • More centrally located ground glass opacities • Less prominent consolidation • Opacities located in the dependent lung regions
Therapeutic response	• More responsive to prone positioning • Better with surfactant • Less responsive to positive end-expiratory pressure • Higher risk for ventilator-induced injury and barotrauma	• More responsive to alveolar recruitment maneuvers • Worse with surfactant • More responsive to positive end-expiratory pressure • Less risk for ventilator-induced injury and barotrauma
Genetic risk	• Increased level of receptor for advanced glycation endproducts • Popeye domain-containing protein 3 (reduced risk) • 308A allele of TNF (reduced risk)	• Increased angiopoietin-2 and von Willebrand factor • Fatty acid amide hydrolase (increased risk) • TNFB22 allele of TNF (reduced risk) • Reduced expression of apoA-IV, C-II, B-100
Outcomes	• Increased mortality • Mild quality of life impairments	• Severe quality of life impairments

### 2.3 Diagnosis

No single diagnostic test confirms or refutes a diagnosis of ARDS. As shown in **Table 3**, the current diagnosis of ARDS is based on the Berlin Consensus Criteria in 2012 according to the timing, hypoxemia, positive end-expiratory pressure (PEEP), chest radiograph, and origin of edema. Safe, inexpensive, and point-of-care tools such as ultrasound imaging and pulse oximetry, can be implemented as diagnostic modalities for monitoring pulmonary infiltration and measuring SpO_2_/FiO_2_ ratio, respectively. In 2016, investigators proposed alternative criteria (SpO_2_/FiO_2_ ratio and lung ultrasound imaging) for ARDS diagnosis-the so-called Kigali modification of the Berlin Criteria. There is no biomarker for ARDS recommended in clinical practice. With the evolution of clinical care and increasing recognition of the global burden of ARDS, and the emerging new methodologies, the advent of these new methods (multiomics, big data, systems biology, and multiplexed point-of-care testing) combined with insights into biomarker strategies will likely yield robust information that will address the dynamic and complex interaction between risk factors, phenotypes, endotypes, and expression modulators and facilitate the development of precision medicine for ARDS patients through the endotype-/theratype-driven approach (**Table 3**).

**Table 3:** Current ARDS criteria and future direction.

	Current Berlin criteria (7) (2012)	Short-term possible revisions (2023+) (19)	Future areas of research (onwards) (19)
**Timing**	Acute onset (<1 week)		Methodologies (big data, systems biology, multiomics, biomarker development, and point-of-care testing development, etc.) addressing the dynamic and complex interaction between risk factors, phenotypes, endotypes, and expression modulators to facilitate the development of precision medicine through the endotype-/theratype-driven approach
**Hypoxemia**	The Berlin definition: PaO_2_/FiO_2_ ≤ 300 Mild: ≤ 201-300 (PaO_2_/FiO_2_) Moderate: ≤ 101-200 (PaO_2_/FiO_2_) Severe: ≤ 100 (PaO_2_/FiO_2_)	SpO_2_/FiO_2_ ratio	
**PEEP**	≥ 5 cmH_2_O	High-flow nasal oxygen, no requirement for minimum PEEP	
**Chest imaging**	Bilateral infiltrates (chest X-ray or CT scan)	Ultrasound	
**Origin of edema**	Not fully explained by cardiac failure		
**New methodologies** • *Biomarkers (plasma and BALF) (* 20–23) ➢ Inflammation (IL-6, IL-8, sTNF-R1, IL-1β, IL-1R, IL-18, HMGB1, NETs, C3a, C4d, C5a, C5b-9, Bb) ➢ Epithelium (RAGE, SP-D, KL-6, CC16) ➢ Endothelium (vWF, Ang-2, ICAM-1; syndecan, endocan) ➢ Coagulation/fibrinolysis (protein C, PAI-1, TM) ➢ Apoptosis (Fas and Fas-L) ➢ Fibrogenesis (laminin, elastin, desmosine, MMPs) • *Multiomics* • *Systems biology* • *Multiplexed point-of-care testing*	None	None	

Ang-2, angiopoietin 2; BALF, bronchoalveolar lavage fluid; CC16, Clara cell secretory protein; Fas, tumor necrosis factor receptor superfamily member 6; Fas-L, Fas ligand; HMGB1, high mobility group box nuclear protein 1; PaO2, partial pressure artery oxygen; FiO2, fraction of inspired oxygen; ICAM-1, intercellular adhesion molecule 1; IL, interleukin; IL-1RA, interleukin-1 receptor antagonist; KL-6, Krebs von den Lungen-6; NETs, neutrophil extracellular traps; PAI-1, plasminogen activator inhibitor 1; PEEP, positive end-expiratory pressure; RAGE, receptor of advanced glycation end products; SP-D, surfactant protein D; SpO2, oxygen saturation; sTNF-R, soluble tumor necrosis factor receptor; TM, thrombomodulin; TNF, Tumor necrosis factor; vWF, von Willebrand factor.

### 2.4 Treatment and management

ARDS requires urgent intubation and ventilation, careful assessment and treatment of the underlying causes and continual reassessment for potential complications. The major treatments are divided into supportive managements and specific managements. Supportive therapies include venous thromboembolism (VTE) prophylaxis, nutrition, mobilization, and sedation. Specific managements comprise lung protective mechanic ventilation, fluid-conservative management, neuromuscular blockade, prone positioning, and extracorporeal membrane oxygenation (ECMO) (24). Despite these standards of care, ARDS is still associated with poor clinical outcomes with a mortality rate of approximately 40%. The lack of effective pharmacotherapies presents a continuing challenge in the field.

## 3 Complement system

The complement system is an integral component of the innate immune system and a major initiator of inflammatory response. It has key roles in the recognition and elimination of invading pathogen-associated molecular patterns (PAMPs) and damage-associated molecular patterns (DAMPs) by enhancing inflammation, opsonization, phagocytosis, the formation of neutrophil extracellular traps (NETs), and cellular lysis *via* bridging innate and adaptive immune immunity. However, exaggerated complement activation can contribute to tissue damage and organ dysfunction.

### 3.1 Key complement components and complement regulators

The complement system has key roles in both the innate and adaptive immune responses. It is composed of nearly 60 complement proteins that interact in a proteolytic cascade. As shown in **Table 4**, complement proteins can be divided into two broad categories: complement components that are involved in activation and complement regulators that inhibit complement activation. Traditionally, three different initiation pathways are known to activate the ComC. Complement can also be activated by extrinsic proteases including coagulation cascade (CoaC) proteases and neutrophil-/macrophage-derived proteases. The complement pathways are tightly regulated by complement inhibitors presented in the soluble form (blood) and the solid form (membrane bound).

**Table 4:** Key complement components and their functions.

Classical pathway	
C1q	Binds to Fc region of Ab
C1r	Activates C1s
C1s	Activates C4/C2
C2	Key component of C4b2a
C4	Key component of C4b2a
C4b2a	Actives C3
C1 inhibitor	Inactivates C1r and C1s
C4BP	Blocks C4b2a formation
Factor I/CD46/CR1	Inactivates C4b
CD55	Prevents C4b2a formation
Lectin pathway	
MASP1	Activates MASP2, MASP3, C4, and CoaC
MASP2	Activates C4
MASP3	Activates alternative pathway
MBL	Binds to mannose residues on microbial surfaces
C2	Key component of C4b2a
C4	Key component of C4b2a
C4b2a	Actives C3
Ficolin	Binds to carbohydrates of bacterial surfaces
Collectins	Binds to oligosaccharide structure or lipids of microorganic surfaces
C1 inhibitor	Inactivates MASP-1 and MASP-2
C4BP	Blocks C4b2a formation
Factor I/CD46/CR1	Inactivates C4b
CD55	Prevents C4b2a formation
Alternative pathway	
C3(H_2_O)	Binds to factor B
Factor B	Key component of C3bBb and C3bBb3b
Factor D	Activates factor B
Properdin	Stabilizes C3bBb
C3bBb	Activates C3
C4BP	Blocks C3bBb formation
Factor H	Inhibits C3bBb formation
Factor I/CD46/CR1/CRIg	Inactivates C3b
CD55	Prevents C3bBb formation
Common pathway	
C3	Key component of classical, lectin, and alternative pathways
Extrinsic pathway	
PMN-/MΦ-derived proteases	Activate C3 and C5
TF	Activate C3 and C5
CoaC factors (FIIa, IXa, Xa, XIIa, etc.)	Activate C3 and C5
kallikrein	Activate C3 and C5
FibC (plasmin, fibrin, etc.)	Activate C3 and C5
Terminal pathway	
C5	The first component (C5b) of C5b-9
C6	Binds to C5b and C7
C7	Binds to C8
C8	Bonds to C9
C9	Polymerized to form C5b-9
C3bBb3b	Activates C5
C4b2b3b	Activates C5
CD59	Inhibits C5b-9 formation
Vitronectin/clusterin	Inactivates C5b-9

C4BP, C4b binding protein; CoaC, coagulation cascade; CR1, complement receptor type 1; CRIg, complement receptor of immunoglobulin; Fib, fibrinolytic cascade; MΦ, macrophage; MASP, mannan-biding lectin serin protease; MBL, mannose binding lectin; PMN, polymorphonuclear leukocyte; TF, tissue factor.

### 3.2 Complement activation pathways

The activated complement provides a critical link between the innate and adaptive immune responses. As shown in **Table 4**, the cascade has 4 initiating arms: the classical, lectin, alternative and extrinsic (driven by activated coagulation/fibrinolytic/kinin proteolytic enzymes) pathways. Each pathway is activated in a distinct mechanism. The classical pathway (CP) is initiated by binding pattern recognition proteins (PRPs) to immune complexes (ICs), C-reactive protein (CRP), serum amyloid P (SAP), DAMPs, and/or PAMPs, whereas the lectin pathway (LP) is homologous to the classical pathway and triggered by binding PRPs to exposed aberrant carbohydrates on foreign, damaged or necrotic cells. Unlike the CP and LP, the alternative pathway (AP) amplifies the initial response and maintains a low level of activity by a mechanism known as “tickover” (25). However, the AP is always primed to respond quickly and vigorously to pathogens or injury, and accounts for as much as 80% of C5a and C5b-9 production.

In addition, the extrinsic pathway that activates ComC has been described by Huber-Lang et al. (26). Traditionally, the plasma cascades, including the ComC, coagulation cascade (CoaC), fibrinolytic cascade (FibC), and kinin cascade (KinC), are described as separate cascades. Nevertheless, these systems belong to complex inflammatory networks (27) and exhibit similar characteristics regarding specialized functions of their activators and inhibitors. Expanding evidence indicates multiple interactions among these cascades under physiological and pathological conditions. It has been documented that active components of the CoaC (factor IIa, IXa, Xa, XIa, XIIa) (26, 28), FibC (plasmin) (29–31), and kinC (kallikrein) (32) can cleave and/or activate proteins (C3, C5) of the ComC, leading to the generation of ComC activation products (C3a, C3b, C5a, and C5b-9).

**Figure 2:**
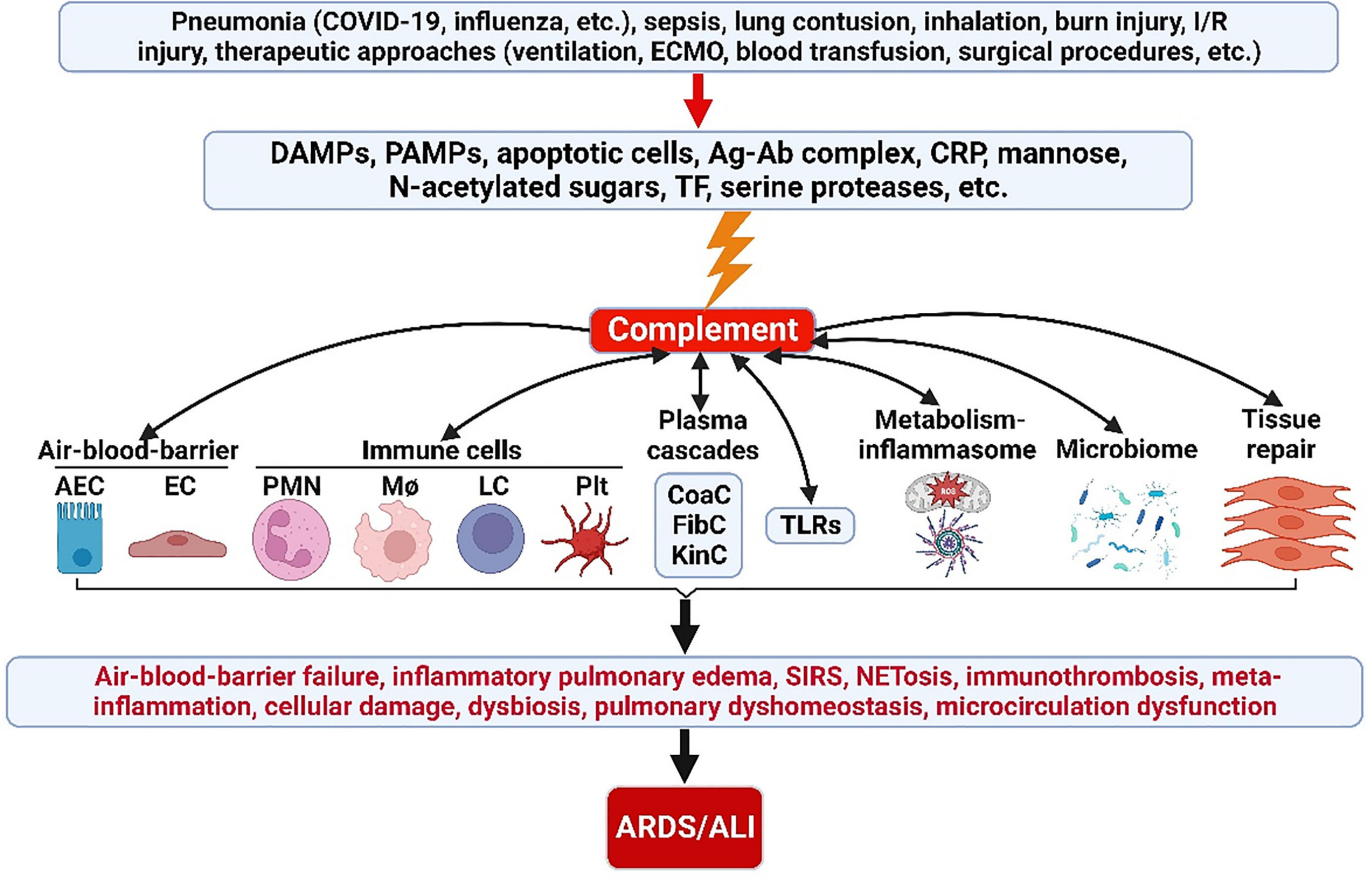
ComC in the pathogenesis of ARDS/ALI. A variety of acute insults trigger complement activation *via* DAMPs, PAMPs, apoptotic cells, Ag-Ab complex, CRP, mannose, N-acetylated sugars, TF and serine proteases. The activated ComC resides upstream of immunity and acts as a vital nexus of the pathobiological connectome for ARDS/ALI. AEC, alveolar epithelial cell; Ag-Ab, antigen-antibody; ALI, acute lung injury; ARDS, acute respiratory distress syndrome; CoaC, coagulation cascade; ComC, complement cascade; CRP, C-reactive protein; DAMPs, damage-associated molecular patterns; EC, endothelial cell; ECMO, extracorporeal membrane oxygenation; FibC, fibrinolytic cascade; I/R, ischemia/reperfusion; KinC, kinin cascade; LC, lymphocyte; NETosis, neutrophil extracellular trap formation; PAMPs, pathogen-associated molecular patterns; PICS, persistent inflammation/immunosuppression and catabolism syndrome; plt, platelet; PMN polymorphonuclear neutrophil; SIRS, systemic inflammatory response syndrome; TF, tissue factor; TLRs, toll-like receptors; MΦ, macrophage.

**Figure 3:**
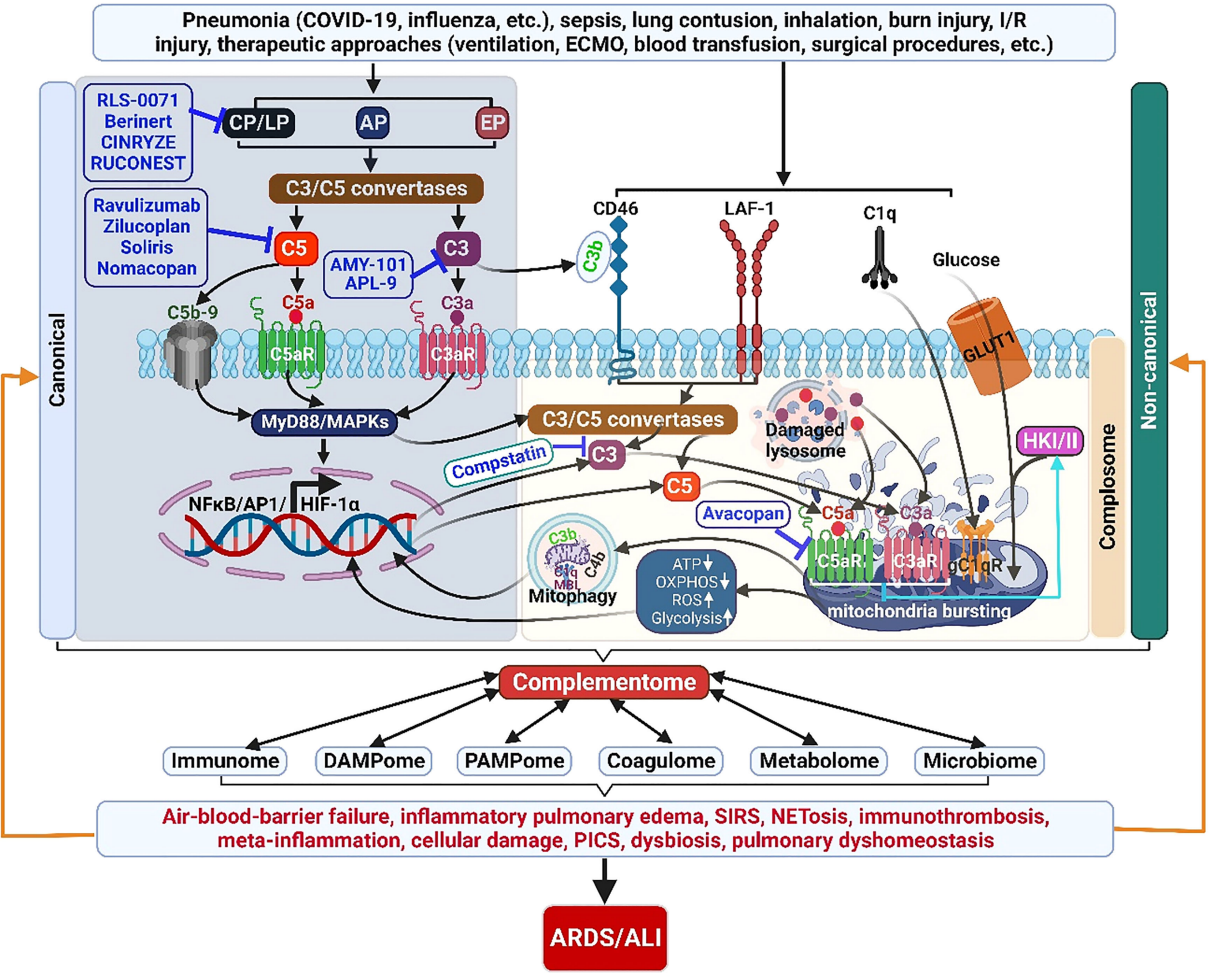
The complementome role and therapeutic targets in ALI/ARDS. Acute insults can induce exaggerated ComC activation locally and systemically. Complement proteins are produced and released intrahepatically providing the systemic extracellular ComC. Complement proteins are also synthesized extrahepatically (locally) by other cells and the intracellular local ComC plays a pivotal role in cell homeostasis and metabolisms. The activated complementome acts as a pivotal nexus to activate other systems of the immunome, coagulatome, metabolome, and microbiome *via* that results in air-blood barrier failure, inflammatory pulmonary edema, SIRS, immunothrombosis, meta-inflammation, PICS, and dysbiosis, ultimately leading to the development of ARDS/ALI. ALI, acute lung injury; AP, alternative pathway; AP1, activator protein 1; ARDS, acute respiratory distress syndrome; C3aR, C3a receptor; C5aR, C5a receptor; CP, classical pathway; ECMO, extracorporeal membrane oxygenation; EP, extrinsic pathway; HIF-1α, hypoxia-induced factor 1-alpha; GLUT1, glucose transporter 1; HKI/II, hexokinase I/II; I/R, ischemia/reperfusion, LAF-1, leukocyte adhesion molecule 1; LP, lectin pathway; NETosis, neutrophil extracellular trap formation; NF-κB, nuclear factor kappa-light-chain-enhancer of activated B cells; OXPHOS, oxidative phosphorylation; PICS, persistent inflammation/immunosuppression and catabolism syndrome; ROS, reactive oxidation species; SIRS, systemic inflammatory response syndrome.

Appropriate activation of the ComC play an important role in host hemostasis, defense against infection, immune complexes and damaged cell clearance, priming adaptive immunity, regeneration, and metabolic reprogramming. However, excessive ComC activation could trigger systemic inflammatory response syndrome, cellular damage, and alveolar vascular/epithelial hyperpermeability leading to ARDS and multiple-organ failure (**Figures 2**, **3**).

## 4 ComC as a pivotal integrator for ARDS pathobiological connectome

### 4.1 Complement in lung homeostasis

The ComC is part of the innate sensor and effector systems that are considered the host’s ´first line of defense´ with a number of biological functions. An intact ComC plays an integrative role in host defense and homeostasis by interacting with microorganisms, removing damaged cells, crosstalking with toll-like receptors (TLRs), bridging with innate and adaptive immune cells, interplaying with the CoaC, promoting tissue repair, and regulating inflammatory resolution. The pulmonary airway is exposed to inhaled pathogens and allergens on a daily basis: Airway epithelial cells and macrophages play a central role in this defense against pathogens and allergens *via* various host mediators including the ComC. Although the liver is the primary site for the synthesis of circulating complement proteins (except C1q, C7, and factor D), various studies in both animals and humans have demonstrated that alveolar epithelial cells, airway epithelial cells and alveolar macrophages synthesize a number of complement components. In particular, alveolar type II epithelial cells produce complement proteins C2, C3, C4, C5, C6, C7, C8, C9, factor B, factor H, factor I, and C1s inhibitor (33, 34), whereas human bronchiolar epithelial cells [C3, Decay-accelerating factor (DAF), membrane cofactor protein (MCP), and CD59] and lung fibroblasts (C1s, C3, C4, C5, C6, C8, and C9) can synthesize complement proteins (34, 35). Alveolar macrophages have the potential to synthesize the complement proteins of the functional alternative and terminal pathways (36), whereas the alveolar macrophages under a disease condition produced more complement components than their healthy counterparts (37). Furthermore, alveolar macrophage-derived serine proteinases cleaved local synthesized C5 into C5a, which initiates pulmonary inflammation (38). This local production not only significantly contributes to the systemic pool of complement, but also forms fully functioning complement pathways in the pulmonary niche thereby influencing local complement processes. The pulmonary synthesis of complement proteins in the microenvironment plays an important role in host defense against infection, clearance of ICs and damaged cells, and regulation of metabolism. Researchers reported that C1q and mannan-binding lectin (MBL) bound to and enhanced apoptotic cell uptake by alveolar macrophages *in vivo* (39). Chang et al. revealed that MBL was present in the lung of healthy wild type mice and MBL deficiency increased susceptibility to influenza A virus infection (40). However, excessive local ComC activation increases pulmonary vascular permeability, initiates thromboinflammation, and causes pneumonic cell damage. Our previous studies have demonstrated that excessive pulmonary complement synthesis and ComC activation contribute to pulmonary inflammation and ALI in a swine model of traumatic hemorrhage (41–44). Furthermore, one would expect prolonged activation by C3a and C5a under such pathological conditions because of the absence of carboxypeptidase N (a major anaphylatoxin inactivator) in the pulmonary interstitial space (45).

### 4.2 Evidence and mechanism of ComC dysregulation in ALI/ARDS

Accumulating evidence is showing that overwhelming or deregulated complement activation may fuel a cytokine storm, endotheliopathy and neutrophil extracellular traps (NETs)-driven thromboinflammation leading to collateral tissue damage. In patients, complement activation has been detected in plasma (46–49) and bronchoalveolar lavage (BAL) fluid (47, 50, 51), as demonstrated by C3a, C3c, C5a and C5b-9 increase. Besides C5a, C3a and C3adesArg (a variation of C3a that plays a central role in the metabolism of adipose tissue) were also elevated in the plasma and BAL fluid obtained from patients with ARDS compared to that obtained from patients that did not develop ARDS (6, 47, 52). However, it seems that C5adesArg was not significantly changed during ARDS (6). In addition, Zilow and colleagues reported a more sensitive assay by monitoring the C3a/C3 ratio in the plasma and BAL fluids to predict the risk to ARDS at an early stage (53, 54). Furthermore, in a particular subtype of ARDS patients, such as trauma related patients, complement activation was also detected *via* increased levels of C3a and C4a in plasma (4, 55). Fosse et al. suggested that different types of ARDS may have different patterns of complement activation (5). In trauma associated patients, complement activation occurred immediately after the injury and correlated strongly with severity scores of lung injury, whereas other types of ARDS (such as in sepsis patients), complement activation occurred at a relatively later stage (5). A recent study demonstrated widespread complement activation characterized by excessive C3a generation, C5b-9 formation, and C3d, C4d, MASP-2 deposition in lung tissues as well as prominent increase in blood C5a in proportion to the severity and high expression of C5aR in blood and pulmonary myeloid cells in patients with severe COVID-19 (56–59). These insights suggest that blockade of the ComC could be used to prevent the neutrophil/monocyte infiltration, endotheliopathy, and thromboinflammation associated with ARDS.

However, the mechanism of ComC activation in ARDS/ALI is largely unknown. Recently, compelling evidence reveals that DAMPs and PAMPs are activators of the ComC in other diseases and conditions. Ratajczak et al. demonstrated that high mobility group box protein 1 (HMGB1) and S100 trigger the complement lectin pathway activation *via* binding to the MBL during mobilization of hematopoietic stem/progenitor cells (60). Similarly, HMGB1 release activated the classical pathway by binding to C1q in a mouse model of brain ischemia/reperfusion injury (61). Furthermore, pattern-recognition molecules such as MBL, ficolins, and collectins binding to invading pathogens or damaged cells initiate ComC activation *via* the lectin pathway (62–65). Therefore, we postulate that the release of DAMPs and/or PAMPs after ARDS/ALI may activate the ComC. Another important factor in regards to ComC activation in the setting of ARDS/ALI is respiratory acidosis-induced ComC activation. Low extracellular pH favors the activation of the ComC and CoaC (66–73). Respiratory acidosis and metabolic acidosis (ischemic conditions) significantly lower the local and/or systemic pH and therefore might lead to complement activation.

To study the pathogenesis of ARDS, animal models are an appropriate approach. No single model can mimic all the pathophysiological changes that are observed in patients (74), but complement activation associated with ARDS development have been explored in several experimental animal models, including related to conditions of sepsis (75), IgG immune complex (76), blunt chest trauma (77, 78), transfusion-related acute lung injury (TRALI) (79) and influenza H5N1 viral infection induced-ALI/ARDS (80). Besides the correlation of complement activation with ARDS development, several other studies involving complement-mediated ARDS pathogenesis have been conducted in animals. Till et al. demonstrated that intravenously injected cobra venom factor (CVF) induces lung injury in rats (81). While direct administration of C5a has been shown to induce alveolar inflammations in guinea pigs (82), rats (76) and rabbits (83). Furthermore, using genetic manipulation of ComC, studies have showed reduced lung damage *via* complement-mediation: SARS-COVID-infected C3^-/-^ mice had reduced pulmonary infiltration of neutrophils and monocytes, reduced systemic inflammation, and less respiratory dysfunction (3); C5^-/-^ mice exhibited a reduction of lung injuries in an ICs-induced ALI model (84); C5aR^-/-^ and C5L2^-/-^ mice had attenuated lung injuries in three models of acute lung injury induced by LPS, IgG immune complexes, C5a, and bacterial polyphosphates (85, 86); and significantly increased survival, increased release of interferon- γ, and decreased production of IL-10 accompanied by improved pathogen clearance were observed in mild-moderate septic models of C5aR1-deficient mice (87). Additionally, pharmacological inhibition of complement levels and complement activation in preclinical and clinical ALI/ARDS demonstrated beneficial effects: C3 inhibition with AMY-101 in ex vivo whole blood infection models reduced IL-6 release (88); anti-C5 antibody (eculizumb) treatment attenuated ALI in a non-human primate septic model (89); C3 inhibitor (AMY-101) was safe and associated with a favorable clinical outcome in a patient with COVID-19 pneumonia with systemic hyperinflammation (90); anti-C5a antibody (IFX-1) treatment led to increased lung oxygenation and decreased systemic inflammation in COVID-19 ARDS patients (58, 59), attenuated ALI in patients with influenza H7N9 viral infection (91); and administration of IFX-1 in patients with severe sepsis and septic shock selectively neutralized C5a without detectable safety issues (92). E culizumab treatment in severe COVID-19 patients with pneumonia/ARDS led to a significant drop in inflammatory markers and duration of disease (93). Taken together, these studies demonstrated that complement activation is associated with ARDS development in both patients and in animal models.

### 4.3 Complement function in the pathogenesis of ARDS

Excessive complement activation in ARDS can be detected very early in both plasma and pulmonary tissue (49, 94–97). However, the mechanism of initiation of complement activation in ARDS is unclear. Several possible mechanisms have been proposed: (1) classical pathway activation *via* C1q binding to the antigen-antibody complex, CRP, SAP and apoptotic cells (98); (2) lectin pathway activation through MBL interaction with PAMPs (for example, the spike protein of SARS-CoV-2, viral RNA, LPS, and lipoteichoic acid) (99); (3) alternative pathway activation by means of DAMPs-TLRs (88).

Upon activation, complement products induce a profound inflammatory response, of which the main contributors are anaphylatoxins C3a and C5a. In physiological conditions, C3a and C5a are rapidly degraded by enzymatic cleavage of the C-terminal arginine residue by plasma carboxypeptidase N and R to generate cleavage products C3adesArg and C5adesArg (100). These cleavage products are involved in ARDS pathogenesis as described by Huber-Lang et al. (6), although the anaphylactic activity of C5adesArg and C3adesArg seems much lower. In the setting of ARDS, C3a interacts with its receptors C3aR, while C5a engages with its two receptors, the C5aR1 (101) and C5L2 (102) to sense the DAMPs/PAMPs signals. These receptors are highly expressed on neutrophils, macrophages, and lymphocytes. All these inflammatory cells are significantly activated in the lungs and are effector cells which perform their biological roles *via* multiple mechanisms. Furthermore, excessive C5b-9 formation can directly damage pulmonary cells, including alveolar epithelial cells and endothelial cells during ARDS as described below.

#### 4.3.1 Complement-mediated alveolar-capillary barrier dysfunction

Profound pulmonary edema and alveolar-capillary injury are major phenomena of the pathophysiological alterations associated with ARDS/ALI. Alveolar-capillary barrier dysfunction in ARDS/ALI highlights the pathophysiological role of the alveolar-capillary barrier in gas exchange, pulmonary fluid balance, immune cell trafficking, inflammatory mediators, noninflammatory signaling, mechanical injury, and lung repair (103). Alveolar epithelial cells (AEC) and pulmonary endothelial cells synthesize most of the ComC proteins (98, 104), so they not only significantly contribute to the systemic pool of complement but also form fully functioning complement pathways in the pulmonary niche, thereby contributing to alveolar-capillary barrier dysfunction if the ComC in the pulmonary system is excessively activated. As a result, increased bronchoalveolar lavage (BAL) fluid levels of C3a were observed in ARDS patients (105). C5a and C5b-9 potentiated alveolar-capillary barrier dysfunction in experimental rodent ARDS models (16, 106), whereas inhibition of C5a-C5aR interaction reversed alveolar epithelial barrier dysfunction and inflammation in a preclinical animal ALI model (107). Moreover, the COVID-19 spike protein can directly activate the lectin pathway by binding to MASP-2 (88). There was substantial deposition of activated complement products (C5b-9, C4d, MASP-2) and their co-localization with COVID-19 spike glycoproteins in the microvasculature of interalveolar septa, and pulmonary microvascular injury was associated with elevated plasma C5a and endothelial C5b-9 deposition in COVID-19 patients with ARDS (57), suggesting a key role of complement-mediated catastrophic microvascular injury syndrome in the pathogenesis of severe COVID-19. Furthermore, C5b-9 formation can cause pulmonary endotheliopathy by activating two independent molecular pathways: the inflammatory molecular pathway and the microthrombotic molecular pathway. This occurs as C5b-9 triggers inflammatory cytokine release and promotes either exocytosis of large von Willebrand factor multimers in the case of the inflammatory nuclear pathway or promotes platelet activation in the case of the microthrombotic pathway (108). More importantly, treatment with a lectin-pathway inhibitor (narsoplimab) in 6 COVID-19 patients with ARDS resulted in a rapid and sustained reduction of circulating endothelial cell counts and serum cytokines and an increase in survival (109). It appears that complement activation participates in regulating alveolar-capillary functions. The mechanism by which the ComC induced pulmonary endotheliopathy remains unclear. Given the function of the ComC on the endothelium, neutrophils, monocytes/macrophages, and coagulation, downstream contributors such as the shedding of complement regulatory proteins from pulmonary endothelium (110), C5b-9-mediated endotheliopathy, NETs-induced platelet-leukocyte aggregates, the cytokine storm, tissue factor (TF)-triggered extrinsic CoaC activation could all have played a role (88, 111).

#### 4.3.2 Complement and inflammation

##### 4.3.2.1 Complement mediates neutrophil activation in ARDS

Complement-mediated neutrophil activation has been proposed as an important pathogenic mechanism causing acute microvascular lung injury in ARDS, an element of the disease which has been extensively studied (11, 112, 113). Complement activation generates anaphylatoxins C3a and C5a. Engagement of C3a and C5a with their receptors on neutrophils leads to neutrophil activation. Activated neutrophils are capable of ingesting microorganisms or particles (phagocytosis), and releasing an assortment of proteinases through degranulation and generation of neutrophil extracellular traps (NETs) composed of chromatin and serine proteases (114). Severe pulmonary neutrophil activation and infiltration lead to lung injuries (95, 115). This pathway explains the major mechanism for alveolar epithelial and capillary endothelial injuries, as evidenced by several studies: 1) Neutrophils were the dominant inflammatory cell type recruited into the lungs during ARDS development, particularly in the acute phase (13, 116, 117); 2) C5a administration to animals led to induction of rapid influx of neutrophils into the lung, and degree of neutrophil activation was associated with disease severity during ARDS (81, 83); 3) The neutrophil swarming in the lungs was largely reduced by blocking C5 interaction with C5aR1 or C5L2; and 4) The induction of inflammatory neutrophils in lungs was reduced greatly and the pathologies were alleviated synchronously in C5aR or C5L2 knockout mice (78, 118, 119).

Neutrophils are granulocytes that contain various biological mediators in their granules. Upon activation, several inflammatory mediators are released into the surrounding milieus, including proteases, reactive oxygen species (ROS), pro-inflammatory cytokines, and pro-coagulant molecules. During ARDS, these molecules contribute to increased vascular permeability and a sustained loss of normal epithelial and endothelial barrier functions (120–122). In addition, recent studies have reported that complement activation-mediated lung injury are *via* NETing neutrophils and necroinflammation (49, 123) and the effectors of extracellular histones (115, 124, 125) in ARDS patients and preclinical animal ALI. It has been proposed that C5a activates neutrophils to form NETs, and that NETs release extracellular histones to exert biological effects. A key observation that links C5a with extracellular histones is that, in C5aR^-/-^ and C5L2^-/-^ mice, lung injuries were attenuated, and level of extracellular histones was reduced by more than 90%. This observation indicates that the appearance of extracellular histones was C5a receptor-dependent (85). In the same experimental settings, depletion of neutrophils in the wild type mice resulted in a 70% reduction in histone levels in BAL fluid (85) suggesting that C5a induced extracellular histone release was largely through neutrophils. Moreover, blockade of C5aR or C3 disrupted NET formation and reduced TF expression in neutrophils (49). In addition, it was reported that circulating histones can rise to toxic levels and serve as mediators of other distant organ damages, although the lung was the organ most susceptible to damage (126). Altogether, current finding indicates that C5a- and C3a-triggered NETosis and extracellular histones release in neutrophils contribute to ARDS development.

In addition, C5a is also reported to cause activation of inflammasomes during ARDS through neutrophils (127). Activated inflammasomes trigger the assembling of the active form of caspase-1. Once active, caspase-1 cleaves cytokine precursors (pro–IL-1β and pro–IL-18) to their mature biologically active and secreted forms (IL-1β and IL-18). Both proinflammatory cytokines IL-1β and IL-18 have been linked to ARDS development (128–130), which indicates that inflammasome activation is a critical factor that contributes to ARDS pathogenesis. Taken together, the aforementioned studies highlight that neutrophil activation by complement pathway is a key mechanism of lung injuries.

##### 4.3.2.2 Complement activates alveolar macrophages

Besides neutrophils, the complement system mediates activation of alveolar macrophages, inducing lung injury. Mounting evidence suggests that macrophages are another major inflammatory cell type activated in the lungs during ARDS, especially in the very early phase. Macrophage activation syndrome is considered to perform a leading role in cytokine storm syndrome during ARDS (131–133). Three classes of macrophages are identified in lung tissue: bronchial macrophages, alveolar macrophages, and interstitial macrophages (134). Of those, alveolar macrophages seem to be paramount, as the alveoli act as the port of entry to the body and are constantly exposed to foreign particles and infectious agents. Alveolar macrophages are the first line of defense against these agents. Findings have shown that alveolar macrophages express high levels of pattern recognition receptors (PRPs) during steady state (135). In addition, the function of alveolar macrophages is highly plastic. Upon stimulation, these macrophages can differentiate into classically activated macrophages (CAMs, also known as M1 macrophages) and alternatively activated macrophages (AAMs, also known as M2 macrophages). M1 macrophages produce high levels of pro-inflammatory cytokines (high levels of IL-12 and low levels of IL-10) in response to paracrine signaling from the Th1 cytokine INF-γ and in response to autocrine signaling by IFN-β, both of which may dominate during the acute phase of ARDS. The M2 macrophages release anti-inflammatory cytokines (high levels of IL-10, TGF-β and low levels of IL-12) in response to signals from Th2 cytokines such as IL-4 and IL-13. Macrophages with the M2 phenotype dominated the resolution phase of ARDS (131).

In the acute phase of ARDS, the resident alveolar macrophages are activated. Meanwhile, more macrophages are recruited into the alveolar compartment and function as pro-inflammatory cells, exacerbating inflammatory response. Kazmierowski et al. reported that during the first four hours of ARDS, alveolar macrophages are the principal respiratory cells (90%) initially recovered from BAL fluid. This indicates that macrophages are first responder inflammatory cells at the onset of lung injury (50). As proposed, anaphylatoxins (C3a, C5a) from complement activation products interact with their receptors (C3aR, C5aR1, C5L2) on macrophages and lead to macrophage activation (95, 98). Activated macrophages are highly phagocytic, and also release effector molecules such as cytokines and chemokines (136, 137), which can rapidly recruit neutrophils into the injury site (138). Activated complement products (C3a, C5a, and C5b-9) also function as “alarmins,” initiating secretion of both IL-1 and IL-18 at NLRP3-, ASC-, and caspase-1-dependent manner in macrophages *via* two signal pathways: (1) the priming signal, triggered by the signal axis of C3a/C5a-C3aR/C5aR-MAPKs-NF-kB/AP-1, leading to the transcriptional upregulation of canonical and non-canonical NLRP3 inflammasome components (139–141); and (2) the activation signal, which is elucidated through the cascades of C3a-C3aR-ERK1/2-ATP-P2X7, C5a-C5aR-mitochondria- ROS, and C5b-9-ATP influx/K+ efflux (140–144). Genetic deletion of C6 in mice resulted in a deficiency of NLRP3 inflammasomes in macrophages and neutrophils that resulted in less lung damage and improved survival after sepsis (145).

Rosseau et al. showed BAL fluid from ARDS patients with a predominant monocyte-like macrophages. These macrophages undergo phenotype switching during the disease progress in sepsis-, pancreatitis-, or severe pneumonia-induced ARDS (146). In a murine model of intestinal ischemia-induced ALI, Hu et al. observed that the binding of C5a produced during ALI to C5aR was increased on alveolar macrophages, leading to apoptosis of alveolar macrophages and development of ALI (147). C5a also induced ALI through STAT3-mediated macrophage activation after intrapulmonary deposition of IgG immune complexes in a rat model (148). Moreover, inhibition of C5a markedly diminished macrophage infiltration into lungs and reduced lung injury (91). This suggests that interruption of C5a/C5aR interaction reduces the infiltration of macrophages, and in turn ameliorated the disease in ARDS. Taken together, these data demonstrated that C5a/C5aR activates macrophages that play a pivotal role in lung tissue damage during ARDS. Besides direct interaction of C5a and C5aR on macrophages, the C5a-C5aR axis also indirectly stimulated alveolar macrophages to release TNFα and neutrophil accumulation in the lungs *via* Fc gamma receptors (FcγR)-mediated signal pathway (149).

On the other hand, macrophages also participate in anti-inflammatory and tissue-reparative phenotypes during the course of inflammation. Steinberg et al. characterized the alveolar inflammation in ARDS patients with multiple types of causes in survivors and non-survivors. They found that alveolar macrophages are increased in survivors of ARDS in BAL fluid. This indicates that macrophages provide a protective function during ARDS, and normal resolution of alveolar inflammation is associated with a favorable outcome (150). In addition, emerging evidence showed that macrophages play a role in anti-inflammation by producing anti-inflammatory cytokines (IL-10) *via* transcription factors (STAT3 and AP-1) (148, 151, 152). However, it is unclear whether the complement - for example, *via* the C5a-C5L2 or C5a-C5aR1 axis - participates in regulating the later phase of inflammation resolution.

##### 4.3.2.3 Complement mediates lymphocyte activation

The complement system is part of the innate sensor and effector system. Accumulated evidence indicates that the complement system functions as a bridge between innate and adaptive immunity (153). Lymphocytes are considered to play major roles in adaptive immunity by inducing immune responses to protect the host. Likewise, lymphocytes highly affect disease progress and outcome during ARDS. In a mouse model, rapid influx of lymphocytes into the lung was observed during the early phase after LPS-induced lung injury. In an LPS induced lung inflammation model, Harris et al. reported an elevation of lymphocyte number over days 3-6 post LPS instillation (154). Morris et al. in an endotoxin induced lung injury model reported that the percentage of lymphocytes in the BAL fluid increased from 1.8% on day 1 post-endotoxin to more than 11% on days 3 and 5 (155). Nakajima et al. also reported a significant increase in CD4^+^ T cells in an LPS-induced ALI model (156). In addition, the indirect role of cytokines produced by lymphocytes contributed to ARDS pathogenesis. Cytokine IL-17A was the most characterized. Evidence from multiple studies supports the view that pro-inflammatory cytokine IL-17A contributed to ARDS pathogenesis (157–159). IL-17A enhances neutrophil recruitment, and also promotes production of inflammatory cytokines. CD4^+^ T cells, γδ T cells (160), and pulmonary innate lymphoid cells (ILC3s) (161) were the major source of IL-17A. Studies revealed that regulatory T cells (Tregs) also contribute to ARDS outcome. The Treg/CD4+ T cells ratio (162) and Th17/Treg ratios were also considered predicative parameters for ARDS patients (163). However, this point remains controversial because recent studies in COVID-19 ARDS patients demonstrated CD4+ lymphopenia (164, 165).

Although the ComC is considered to bridge the innate and adaptive immune systems, it remains largely unknown how the complement system regulates lymphocyte activation during ARDS. T cells and antigen-presenting cells (APCs) produce complement proteins and yield local complement-activated products, C3a and C5a. These anaphylatoxins can interact with C3aR and C5aR expressed on the T cells and APCs including dendritic cells and macrophages. These interactions trigger a series of events including recruitment of T cells, enhancement of T cell proliferation, prevention of T cell apoptosis (163, 166), release of cytokines (IL-12, IL-23), and upregulation of co-stimulatory molecules CD80 and CD86 (167). Genetic and pharmacological manipulation of complement activation attenuated T cell-mediated immunity and delayed allograft rejection in mice (166, 168). These findings suggest that C3a and C5a provide a vital bridge between innate and adaptive immunity, extending the roles of C3a and C5a in inflammation. Besides effector T cells, complement may also modulate other types of lymphocytes, Tregs and γδ T cells. Studies demonstrate that Treg subsets contributed to the resolutions of LPS-induced ALI, as reported by D’Alessio and colleagues (169). Significant accumulation of γδ T cells in lungs was observed during CLP-induced ALI (167). Complement C4 induced Treg differentiation *via* dendritic cells was observed in systemic lupus patients (170). However, there is a lack of experimental support to determine whether complement is involved in regulating these lymphocytes.

##### 4.3.2.4 Complement-mediated platelet activation

In addition to clot formation and homeostasis, platelets also play an essential role as immunological multitaskers. Platelets contain complement factors and bear complement receptors, and can activate the ComC and vice versa. The ComC and platelets cooperate against pathogens and are involved in inflammatory diseases (171). Platelets are a key factor in the pathophysiology of ALI *via* the DAMPs/PAMPs-TLRs axis and platelet-neutrophil interaction at the site of injured endothelium (103). Increasing evidence demonstrates the role of platelets in and their interaction with the ComC in tissue inflammation and organ damage (172). Activated platelets potentiated MASP1/2 activation of the complement lectin pathway in thromboinflammation (173). C5a-dependent up-regulation of lung vascular P-selectin was reported *in vitro* and *in vivo* studies (174). Platelet-derived microparticles induced ComC activation (175) and activated platelets amplify complement-mediated lung endothelial damage by releasing serotonin, which in turn potentiates granulocyte adhesion to pulmonary endothelium (176). Mannose-binding lectin acted as an early trigger of platelet activation and vascular damage after cerebral ischemia in mice (177). Inhibiting recruitment of platelets at the sites of injured endothelial cells attenuated complement activation and lung injury in an ex vivo perfusion of porcine lung (178). It seems that complement-platelet interaction may participate in the pathophysiology of ARDS/ALI.

The emerging understanding of the pathogenic mechanisms underlying SARS-CoV-2 infection and COVID-19 progression highlight the critical role of complement-mediated neutrophil and platelet activation, endotheliopathy, and thromboinflammation (96). Recent studies in COVID-19 further demonstrate that crosstalk between complement and coagulation functions as a key trigger of COVID-19-induced ARDS. Indeed, COVID patients demonstrated systemic complement activation, which was associated with pulmonary thromboembolic events and disease severity (48, 179). Skendros et al. reported a pivotal role of complement activation in COVID-induced immunothrombosis *via* C5a-C5aR-NETs/TF or C3a-C3aR-thrombin-NETs/TF, while pharmacological blockade of C5aR1 or C3 disrupted NETosis-/TF-driven thrombogenicity (49).

#### 4.3.3 Cross-talk between complement cascade and other plasma cascades in ARDS

Plasma contains four enzymatic systems: ComC, CoaC, KinC, and FibC. All relate to produce the inflammatory response. Emerging data indicate that interplay among plasma cascades fuels hyperinflammation and thrombotic microangiopathy (thromboinflammation), thereby increasing lung injury and mortality. One of the primary pathologies in ARDS is thrombogenesis as indicated by the florid fibrin deposition in the intra-alveolar space and microthrombi in the pulmonary microvasculature. Profound fibrin deposition and microthrombi in ARDS likely result from both activation of the CoaC and impairment of the FibC (180, 181). Fibrin deposition and the associated dysfunction of the FibC were evidenced from clinical studies in ARDS. Fuchs-Buder et al. found that the pro-coagulant pathway and fibrin degradation are markedly activated in ARDS patients compared to non-ARDS patients (182). Tomashefski et al. observed thrombo-emboli and macrothrombi in patients with ARDS (183). Bone et al. also found that fibrin microthrombi were present in the lungs of a majority of ARDS patients (184). The latest studies observed that hypofibrinolysis was related to increased plasminogen activator inhibitor-1 (PAI-1) presence in COVID-19 patients (185). Interestingly, increases of both tissue plasminogen activator and PAI-1 in all COVID-19 patients regardless of disease severity suggest that severe SARS-Cov2 infection may hijack normal profibrinolytic signaling and lead to a procoagulant state, including increased PAI-1 expression, prolonged PAI-1 half-life, and severe lung injury (185, 186). In addition to that, thrombocytopenia was observed in several studies and associated with ARDS mortality (187–189). Moreover, recently Lipcsey et al. presented evidence that the ComC and the KinC were strongly activated and associated with lung damage, illness severity score and mortality in critically ill patients with COVID-19 (190).These studies highlight that plasma cascades participate in ARDS development and highly affect the outcomes of ARDS.

The cross talk between the ComC and the other plasma cascades is reciprocal. In one way, during the course of inflammation, complement effectors can directly enhance coagulation system; specifically, complement works with inflammatory mediators to increase the thrombogenicity in blood. For example, in the setting of ALI/ARDS, Kambas et al. observed that C5a works with inflammatory cytokines, particularly TNF-α, to promote coagulation activation and can stimulate neutrophils from patients to produce TF resulting in the formation of hyaline membranes in the lungs of patients (191). In addition, complement can inhibit anticoagulant factors. Studies showed that C5a may stimulate PAI-1 production from mast cells and basophils (192, 193). PAI-1 is an inhibitor of fibrinolysis, the physiological process that degrades blood clots. Coagulation enzymes also regulated complement pathway activation. Huber-Lang et al. in a sepsis model showed thrombin can directly serve as C5 convertase to directly activate the complement pathway in the absence of C3 (26). Moreover, a recent study reported that intensive ComC activation (C3a, C3c and C5b-9) was positively correlated with thromboembolic events in patients with COVID-19 (48). Moreover, the crosstalk between the CoaC and the ComC is also well illustrated by the ability of certain coagulation enzymes to activate complement components (194, 195). Taken together, these insights illustrated that complement and coagulation systems can modulate each other and contribute to ARDS development *via* thromboinflammation and endotheliopathy (**Figures 2**, **3**).

#### 4.3.4 Interaction between complement and toll-like receptors

The lung is continuously exposed to a diverse array of infectious agents, foreign antigens, and host-derived danger signals. Resident macrophages, neutrophils and stromal cells of the lung constitutively express PRRs, which recognize PAMPs and DAMPs. PRRs are comprised of toll-like receptors (TLRs), complement proteins [C1q, CR1, C4BP, propoerdin, factor H, MBL, collectins and ficolins) (196, 197), RIG-I like receptors (RLRs), and NOD like receptors (NLRs).

The ComC and DAMPs are the major components of the innate immune system. However, these two major innate immune arms were described and studied primarily as separate entities. Nevertheless, an emerging body of evidence indicates close interaction and crosstalk between them in regulating the immunological processes, and the dysregulation of any of the two innate immune arms impact the other through synergistic and/or antagonistic interactions that can modulate host immune homeostasis. In particular, several studies of ComC and high mobility group box 1 (HMGB1) crosstalk *via* its receptors (TLRs) have recently reported that (1) C5a stimulated chemokine CXCL-10 generation in osteoclasts *via* the interaction between C5aR1 and TLR2 (198); (2) TLR-4 activation enhanced C5a-induced pro-inflammatory response by inhibiting the second C5a receptor (C5L2) (199); (3) C5a participated in the regulation of TLR4-induced IL-10 and IL-12 production in macrophages in response to TLR ligand stimulation (200, 201); (4) C5L2 promoted NLRP3 (NOD-, LRR- and pyrin domain-containing protein inflammasome activation and extracellular HMGB1 (eHMGB1) release from macrophages (202); (5) eHMGB1 facilitated C5a-primed neutrophil activation (203); and 6) eHMGB1 release resulted in ComC activation through binding to C1q to exacerbate sterile inflammation (61). Furthermore, other clinical data also elucidated that early eHMGB1 release after severe trauma in patients was associated with post-traumatic complement terminal pathway (CTP) activation and tissue hypoperfusion (204). Consistent with these findings, our recent studies have shown that (1) ComC activation (C3a, C5a, C5b-9, Bb) post-trauma positively correlated with plasma levels of eHMGB1 (205) and histone-complexed DNA fragments (hcDNA) in trauma patients (206); (2) blocking C5 by nomacopan reduced the release of eHMGB1 (206), whereas HMGB1 inhibition by CX-01 also reversed ComC activation in a rat model of traumatic hemorrhage-induced ALI (205); and (3) CTP inhibition by DAF reduced expression of HMGB1, RAGE, NF-kB, cytokines (IL-6, IL-12, IL-13, IL-18, GRO KC) and caspase-1, and HMGB1 nuclear translocation in blast-induced ALI in rats (207). Altogether, these findings indicate synergistic crosstalk between the ComC and DAMPs that markedly amplifies pro-inflammatory responses and may contribute to ALI. Indeed, combined inhibition of C5 and TLR co-receptor CD14 had a pronounced effect on attenuating systemic and local inflammatory response, improving pulmonary function and hemodynamics, and reducing morbidity and mortality in murine (208) and swine sepsis models (209–212). Inclusively, these data suggest that the multifaceted activation and interaction of these two cascades are of crucial importance for NI and highlight the complexity of the inflammatory response after ALI. Therefore, dual blockade might provide a general, broad-acting therapeutic regimen against ALI/ARDS where CTP and HMGB1 are improperly activated.

#### 4.3.5 Complement-metabolism-inflammasome axis

Recent insights into ComC-mediated pathomechanisms in multiple-organ dysfunction syndrome uncover its new function in metabolism. Indeed, Denk et al. has recently demonstrated that C5a-C5aR1 engagement after septic shock induces acidosis *via* an increase in glucose uptake and glycolytic influx, and Na+/H+ exchanger activation in neutrophils (213), suggesting that C5a-C5aR1 is able to significantly create a micro-milieu with lactate acidosis features even in the absence of an oxygen deficit (214). Given the aforementioned low pH favors the activation of both the ComC and the CoaC, we speculate that respiratory/metabolic acidosis after ARDS may cause further ComC activation resulting in a vicious cycle with continued metabolic acidosis and inflammation, implicating that C5a may function as a metabolic switch toward acidosis and ARDS.

In addition to its pivotally canonical host protection by the classical liver-derived and blood-effective ComC, unexpected recent findings have revealed that an intracellularly active complement system, the complosome, has emerged as a centrally non-canonical regulator of the core metabolic pathways fueling immune cell activity (**Figure 3**). So far, little is known about the exact mechanism of the complosome in metabolic reprogramming of immune cells in tissues. However, a plausible scheme is that the complosome intercommunicates with other intracellular immune sensors such as inflammasomes and intracellular TLRs (TLR3, TLR7, TLR8, TLR9). Emerging evidence presents a common network of integrin/T cell receptor (TCR)/TLR-complosome-metabolism in the regulation of immune cell function: 1) intracellular complement synthesis and activation is triggered by integrin/TCR/TLR activation/stimulation in immune cells (215, 216); 2) Activated TCR signals shuttle C3a and C3b to the cell surface where they engage surface-expressed C3aR and CD46 respectively (215, 216); 3) C3b-CD46^CYT-1^ signaling triggers three key metabolic events regulating immune cell effector function: an increase in expression of nutrient transporters (GLUT1, LAT1, and CAT1) that allows to promote glucose and amino acid influx as well as late endosomal and *lysosomal* adaptor and mitogen activated protein kinase and *mTOR* activator 5 (LAMOR5)-driven mammalian target of rapamycin complex 1 (mTORC1) assembly at the lysosomes, elevation in expression of metabolic enzymes (fatty acid synthase, GAPDH, etc.), and activation of intracellular C5a-C5aR-mitochondrial ROS-inflammasome axis, which lead to the high levels of glycolysis, oxidative phosphorylation (OXPHOS), ROS production and NLRP3 inflammasome activation, resulting in IFN-γ/granzyme B-mediated CD4^+^ Th1 induction and cytotoxic CD8^+^ T cell activation, and macrophage IL-1β and IL-18 production in tissues (140, 215); 4) in resting immune cells, C3b-CD^46CYT2^ interaction reduces glycolysis and OXPHOS, increases cholesterol efflux and cMaf musculoaponeurotic fibrosarcoma oncogene homolog (MAF) expression, and C5a-desArg-C5L2 engagement and C1q-gC1qR signal reduce intracellular C5a-C5aR1-mtROS-NLRP3 inflammasome activity, which cause IL-10 release/Th1 contraction and decreased IL-1β/IL-18 secretion respectively (215); 5) Intracellularly generated C3a binds to intracellular C3aR on the lysosome and sustains T cell survival in the tissue through tonic mTOR activation (215). However, a mechanistic explanation for complosome-metabolism in ALI/ARDS still remains elusive and needs further research.

#### 4.3.6 Complement in lung microbiome dysbiosis

The lung microbiome symbiosis maintains homeostasis of the lung’s immune system. Dysbiosis of gut microbiota contributes significantly to the pathogenesis of MOF including ARDS. The lung and gut microbiomes undergo profound interchangeability in critically ill patients such as those with ARDS. Indeed, compelling evidence has revealed that early lung microbiome dysbiosis characterized by the translocation of gut-associated microbes was found to correlate with alveolar and systemic inflammation and lung injury in critically ill patients with ARDS, sepsis, mechanical ventilation, trauma, ischemia-reperfusion injury, hyperoxia and aspiration (217–220). The pathogenesis of lung microbiome dysbiosis in ARDS, however, is largely unexplored. Emerging findings of the lung microbiome and its alteration in ARDS has broadened a new model of pathogenesis: an interaction network between exposure (such as sepsis, mechanical ventilation, trauma etc.), lung microbiome, and host (221). The host’s innate immune system (including complement) plays a critical role in the detection and clearance of bacteria, and complement-based recognition of pathogens or PAMPs initiates inflammation and tissue damage. Recently, there is emerging evidence that (1) SARS-CoV-2-associated ARDS in severe COVID-19 patients directly and indirectly activated the complement cascade (88, 111); (2) complement was found to participate in inflammatory tissue damage *via* dysregulation of host-microbiome homeostasis of the skin and periodontium (222, 223); (3) glycan of the gut fungal wall binding to MBL activates the ComC (224); (4) C5 activation induced diabetic kidney disease and C5aR blockade alleviated renal function and inflammation *via* partly reversing the declines of gut microbiota diversity/abundance and gut levels of short-chain fatty acids (225); (5) microorganisms frequently “highjack” complement proteins (C3d, C3b, iC3b, qC1qR, C3, C5) to gain entry into immune and non-immune cells (226); and (6) interaction between complosome and intracellular pathogens triggers mitochondrial anti-pathogen signaling which increases the expression of transcription factors (NF-κB and AP-1), which subsequently activate inflammasome and mediate a maladaptive proinflammatory response (IL-1β, IL-6, IL-18, IFN-β) (216, 226). However, the involvement of complement in lung microbiome dysbiosis and their interaction in the pathogenesis of ARDS remains poorly understood. The latest studies have highlighted the emerging important functional connection between the complosome, TLRs, metabolism, and the inflammasome axis in immune cells in regulation of host-pathogen interaction, immune cell activation, inflammation, metabolism and hemostasis (139–141, 215, 216, 226). Given that, undoubtedly it will be of significant interest to investigate the complex interplay of the complosome and lung microbiome dysbiosis and their pathogenesis in ARDS. With further insights, this area of research might lead to the development a novel, microbiome-targeted (synbiotics and fecal microbiota transplantation), and immunomodulation strategies in management of ARDS.

#### 4.3.7 Complement activation in lung tissue repair

The role of ComC activation in the resolution of inflammation and tissue repair has been largely ignored when compared to its inflammatory aspects. In particular, accruing evidence reveals that C3a and C5a have regenerative functions on the liver, neurons, osteoblasts, and dental pulp progenitors (227–232). It is noteworthy to mention that C3a/C5a-triggered homeostasis, tonic inflammation, T cell activation, capillary leak syndrome, and edema are also necessary for the resolution of inflammation, restoration of functional tissue, and the clearance of debris and damaged cells (227, 233–236). C1q, MBL, and complement C3 fragment C3b and its receptors (CRs1-4, CD46) are important to facilitate removal of apoptotic/necrotic cells, ICs, and pathogens by enhancing phagocytosis of monocytes and macrophages and T cell homeostasis (140, 237–240). Langlois et al. measured the levels of C1rC1s-C1 inhibitor complex and the C3bP and found that the classical and alternative complement pathways are activated one day before its resolution in ARDS patients in addition to the complement activation two days prior to diagnosis of ARDS (241). Complement C3 inhibition by compstatin decreased early fibrogenic events in sepsis-induced ARDS in baboons (242), suggesting that inhibiting complement could be a potential strategy for preventing pulmonary fibrosis in ARDS patients. Furthermore, during the acute phase of ARDS, C5 exhibited a protective and anti-inflammatory role through regulating matrix metalloproteinases (MMP) and cell migration, while C5 displayed a detrimental effect during the chronic phase of ARDS likely *via* increasing expression of fibrogenic transforming growth factor beta1 (TGF-β) and MMP-3, revealing that C5 plays opposing roles in both inflammation and lung tissue repair (243).

In summary, acute insults (pneumonia, sepsis, trauma, inhalation, I/R injury, therapeutic approaches, etc.) with DAMPs/PAMPs can induce exaggerated ComC activation locally and systemically. The activated complementome functions as a crucial nexus to activate other systems of the immunome, coagulome, metabolome, microbiome, lipidome, genome, epigenome, proteome, and neuroendocrinome, which lead to ALI/ARDS *via* complementome-driven/mediated EC/AEC barrier failure, SIRS, immunothrombosis, immunometabolic dysfunction, PICS, cellular damage, dysbiosis, electrophysiological dysfunction, lung dyshomeostasis, and microcirculation dysfunction (**Figure 2**). This new ComC pathomechanism and its interplay with other systems provide insight into the novel development of more effective therapeutic strategies with important implications for clinical translation for ALI/ARDS patients.

## 5 Targeting ComC therapies in ALI and ARDS

ARDS is present in >10% of intensive care unit admissions and in nearly 25% of ventilated patients and is associated with a high mortality rate at 40%. Lung-protective mechanical ventilation strategies are still the mainstay of ARDS management. Despite more than 50 years of study, current therapeutic interventions for ARDS remain largely limited to pulmonary-supportive strategies, which fail to address the profoundly destructive impact of unchecked inflammation-driven ALI/ARDS. Despite improved understanding of the biological processes underlying the ARDS, the current lack of FDA-approved pharmacological therapies aimed at the underlying pathobiology for ALI/ARDS is a serious unmet need.

As stated above, complement activation is critically involved in the initiation and progression of ALI/ARDS. Therefore, pharmacological targeting of the ComC as well as molecules contributing to complement activation to harness immunothrombosis, alveolar–capillary barrier function, immunometabolic dysfunction and microbiome dysbiosis represents a highly promising approach for the treatment of ALI/ARDS (**Figure 3**).

### 5.1 Preclinical studies

Complement interventions for ALI/ARDS in preclinical studies are under active investigation. In the context of trauma-, hemorrhage-, sepsis-, virus- and IR-induced ALI/ARDS, the compiled evidence of preclinical experiments focusing on the role of complement in ALI/ARDS is shown in **Table 5**.

**Table 5:** Summary of studies in terms of the role of complement in ARDS/ALI in pre-clinical and clinical studies.

Disease	Species	Therapeutic targets/Mechanisms	Pathway	Findings
TR-ALI (244)	Mouse	C1INH	CP	C1INH attenuated pulmonary C3a and improved lung injury
Hyperoxic ALI (245)	Mouse	C4a	CP, LP	C4a treatment attenuated inflammation and hyperoxic lung injury
IgGICs-ALI (246)	Mouse	Zonulin INH, α-Zonulin	CP, LP	1) zonulin induced generation of C3a and C5a; 2) zonulin inhibitor or α-Zonulin attenuated ALI
Lavage-ALI (247)	Rat	C1INH	CP, LP	Reduced neutrophil infiltration
H-ALI (43)	Swine	C1INH	CP, LP	Reduced lung injury/inflammation
TH-ALI (41)	Swine	C1INH	CP, LP	Reduced lung injury, fluid requirement, and inflammation; Increased survival
LT-ALI (248)	Human	C1INH	CP, LP	Attenuated capillary leak syndrome
Aspiration-ALI (17)	Mouse	sCR1, C3-/-, C4-/-, WT	AP	1) increased C3 deposition on injured alveolar pneumocytes; 2) attenuated lung injury by sCR1 or in C3-/- mice, but not C4-/- mice
IR-ALI (18)	Mouse	CRIg-Fc	AP	CRIg-Fc prevented ALI by limiting C activation and inflammation
H1N1-ALI (249)	Mouse	Coversin (nomacopan)	Terminal	Reduced neutrophil/macrophage infiltration and lung damage
LPS-ALI (250)	Mouse	WT, C5L2-/-	Terminal	More neutrophils, MPO, cytokines in BAL, and more airway edema in C5L2-/- or α-C5L2-treated mice than WT mice
LPS-, IgGICs-, C5a-ALI (85)	Mouse	None	Terminal	Lung injury was linked to C5a-C5aR-extracellular histones axis
LPS-PPA/ALS (251)	Mouse	C5 INH, C5-/-	Terminal	1) α2-receptor-mediated PPA and shock dependent on MΦ and C; 2) LSP-induced PPA & ALS depended on α1- & α2-receptors, MΦ and C
LPS-ALI (252)	Mouse	C5aRα	Terminal	Protected lung injury
Trauma-ALI (253)	Mouse	None	Terminal	Increased levels of C5a in BAL 24-72h after trauma
Trauma + LPS (254)	Mouse	WT, C5-/-	Terminal	Decreased local levels of caspase-3, MPO, cytokines, and increased systemic levels of KC in C5-/mice compared to WT mice
IgGICs-ALI (107)	Mouse	C5aR siRNA	Terminal	1) IgGICs increased C5aR expression on bronchial and alveolar epithelial cells; 2) IgGICs resulted in pulmonary edema and inflammation; 3) silencing C5aR by C5aR siRNA attenuated pulmonary edema and inflammation
IgGICs-ALI (84)	Mouse	WT, C5-/-	Terminal	Greater pulmonary neutrophil infiltration, edema, hemorrhage in C5 sufficient mice
IgGICs-ALI (255)	Mouse	Coversin (nomacopan)	Terminal	Mitigated lung injury
TR-ALI (79)	Mouse	WT, C5-/-, C5aR-/-	Terminal	RALI pathogenesis was C5a and C5aR dependent
IR-ALI (147)	Mouse	None	Terminal	C5a exacerbated ALI *via* C5aR-mediated autophagy-induced alveolar macrophage apoptosis
IR-ALI (147)	Mouse	None	Terminal	C5a exacerbated ALI *via* C5aR-mediated autophagy-induced alveolar macrophage apoptosis
C5a-ALI (16)	Mouse	WT, C5aR-/-	Terminal	The detrimental effects of C5a in this model are partly mediated through CCR5 activation downstream of C5aR1
Blast-ALI (256)	Rat	Coversin (nomacopan)	Terminal	1) Nomacopan treatment decreases local and systemic inflammatory response; 2) attenuates tissue and organ damage: 3) improve survival
Blast-ALI (78)	Rat	α-C5a	Terminal	Mitigated lung injury and inflammation
CVF-ALI (257)	Rat	C3aRα/5aRα	Terminal	Inhibited vascular leakage/neutrophil infiltration
CVF-ALI (119)	Rat	α-C5 Ab	Terminal	Reduced lung permeability/inflammation
RAAA (258)	Rat	α-C5	Terminal	1) RAAA increased lung permeability, MPO, TNF-α Mrna; 2) these increases were attenuated by C5 inhibitor treatment
RAAA-ALI (259)	Rat	C5aRα	Terminal	C5aRa INH protected lung injury and pulmonary inflammation
H7N9-ALI (91)	Monkey	α-C5a	Terminal	1) α-C5a attenuated lung injury, pulmonary infiltration of macrophages and neutrophils; 2) α-C5a decreased SIRS
IR-ALI (260)	ex vivo (rat)	sCR1	All	IR resulted in neutrophil stiffness, lung neutrophil retention, and increased lung permeability, effects that were prevented by sCR1
fMLP-ALI (261)	ex vivo (rabbit)	CLU	All	1) CLU suppressed the PAP and PAW, reduced C5b-9 formation and TXA2; 2) CLU attenuated lung edema
NHS-ALI (262)	ex vivo (rabbit)	C1INH, sCR1	All	Decreased PAP, TXA2, lung edema, C3c and C5b-9 deposition by C1INH or sCR1
Paraquate-ALI (263)	Mouse	WT, C3-/-, CR2-Crry, Coversin, C3Rα C5aRα, CR2-fH	All	1) paraquat increased pulmonary expression of C1q, C3, C3aR, and C5aR; 2) paraqual induced lung injury; 3) C3 deficiency, C3INH, CR2-Crry, or CR2-fH improved survival, pulmonary inflammation, and lung injury
IgGICs-ALI (264)	Mouse	C3-/-	All	IgGICs-induced ALI/inflammation was C3-dependent
IgGICs-ALI (26)	Mouse	C3-/-, C5-/-, WT, ATIII, hirudin		1) IgG-IC-induced ALI was C5a-dependent in C3-/- mice; 2) C5^-/-^, ATIII, or hirudin attenuated lung injury and C5a levels in BAL
IR-ALI (265)	Mouse	DAF	All	DAF inhibited CRP+IR-induced complement activation and ALI
IR-ALI (266)	Mouse	CR2-Crry	All	Mitigated lung injury
Liver injury-ALI (267)	Mouse	CVF	All	C depletion enhanced pulmonary inflammatory response
H5N1 ALI (80)	Mouse	C3aR INH, α-C5a, CVF	All	Attenuated inflammation and lung injury
PNTS-ALI (268)	Rat	IVIG	All	1) decreased binding of the pathogenic antibody to lung tissue and prevented the deposition of C3; 2) attenuated the acute lung lesion
CVF-ALI (269)	Rat	sCR1	All	Reduced lung permeability/inflammation
IR-ALI (270)	Rat	CVF	All	CVF attenuated IR-induced inflammation, lung injury
TH (271)	Rat	WT, TXA	All	1) increased lung edema, inflammation, plasmin activity after trauma + HS; 2) TXA reduced pulmonary C5a levels and lung injury in rats
Blast-ALI (272)	Rat	None	All	Increased pulmonary levels of C3 as early as 10 min after trauma
LPS-ALI (273)	Rat	CAEP	All	1) decreased serum levels of CH50, C3, and C4 in LPS-induced ALI; 2) CAEP improved the levels of complement and lung injury
CVF-ALI (274)	Hamster	NE INH	All	1) increased lung vascular permeability and neutrophil infiltration by CVF; 2) lung vascular permeability & neutrophil infiltration reduced by NE INH
Xenogeneic-ALI (275, 276)	Swine	hDAF/hCD59	All	Prevented lung injury
H-ALI (44)	Swine	DAF	All	1) Increased survival. 2) Reduced lung injury, fluid requirement and inflammation
LT-ALI (277)	Pig to monkey	DAF; C1INH	All	1) DAF prolonged survival, improved oxygenation, reduced lung vascular resistance; 2) DAF attenuated platelet sequestration, lung injury, and C5b-9 pulmonary deposition; 3) C1INH did prolong survival of h-DAF transgenic lungs
LPS-ALI (278)	Mouse, rat, Human	None	All	1) LPS elevated levels of fB, C2, C3, C4, C5, C6 in human BAL; 2) LPS increased levels of C3, C5, and fB in mouse and rat BAL; 3) LPS enhanced mRNA expression of fB, decreased C5 in mouse and rat BAL
Trauma-LPS-ALI (253)	Mouse	WT, Hirudin, α-C5a or C5aRα	Terminal, CoaC,	1) increased levels of C5a in BAL 24-72h after trauma; 2) decreased thrombin in BAL by Hirudin correlated with reduced C5a. α-C5a or C5aRα; 3) α-C5a or C5aRa reduced inflammation and ALI
TR-ALI (279)	ex vivo	None	None	Antibody-induced neutrophil activation, but not complement, as a trigger for TRALI
LPS-ALI (280)	Mouse	C3-/-, C5-/-, WT	None	1) neutrophil-dependent, but not complement-dependent; 2) increased MIF, LTB4, and HMGB1

TR, transfusion related; ALI, acute lung injury; INH, inhibitor; CP, complement classic pathway; sCR1, soluble complement receptor 1; WT, wild type; CRIg, complement receptor of immunoglobulin; LP, complement lectin pathway; IgGICs, IgG immune complexes; C1INH, C1 inhibitor; MPO, myeloperoxidase; BALF, bronchoalveolar lavage fluid; LPS, lipopolysaccharides; PPA, phenylpropanolamine; MΦ, macrophage; ALS, anaphylaxis-like shock; IR, ischemia-reperfusion; CCR5, C-C chemokine receptor 5; Ab, antibody; RAAA, ruptured abdominal aortic aneurysm; SIRS, systemic inflammatory response syndrome; fMLP, N-formyl-Met-Leu-Phe; CLU, Clusterin; PAW, peak airway pressure; TXA, tranexamic acid; NHS, normal human serum; TXA2, thromboxane A2; PAP, pulmonary artery pressure; ATIII, antithrombin III; CRP, C-reactive protein; DAF, decay-accelerating factor; CVF, Cobra Venom Factor; IVIG, intravenous immune globulin; Crry, complement receptor 1-related gene/protein; PNTS, rabbit anti-rat lung serum; TH, traumatic hemorrhage; CAEP, crude Arnebiaeuchroma polysaccharides; NE, neutrophil elastase; H, hemorrhage; LT, lung transplantation; MIF, macrophage migration inhibitory factor; LTB4, leukotriene B4; HMGB1, high mobility group box1.

### 5.2 Clinical trials

Considering clinical trials of ALI/ARDS, 29 observational studies addressed complement activation after trauma, sepsis, viruses, and transplantation (**Table 6**). These clinical observational studies (excluding one) further demonstrated that early complement activation plays a pivotal role in the initiation and development of ALI/ARDS, and is associated with mortality. They also elucidate that complement activation is useful for prognosis and diagnosis of ALI/ARDS (**Table 6**). Further investigation should be carried out to test whether complement targeting pharmacotherapeutic strategies have any benefits in ALI/ARDS patients.

**Table 6:** Overview of observational clinical studies evaluating complement role in ARDS/ALI.

Patients	Participants	Role of complement/References
ARDS	124	BALF levels of sCR1 were significantly increased in ARDS patients (281)
ARDS	36	BALF levels of C proteins were differentially expressed between survivors and non-survivors (282)
ARDS	26	Blood C activation was associated with ARDS pathological mechanism (283)
ARDS	15	Plasma C3a and C4a were helpful in predicting ADRS 16hrs post-injury (4)
ARDS	8	Elevated plasma C3a had better predictive value than WBC counts for ARDS during the first 24hrs (284)
ARDS	10	Plasma and BALF C3 and PFB activation and C5a were increased in ARDS patients (47)
RDS	53	Poor responders to surfactant had lower plasma C4 and C3c at admission and 24hrs after birth (285)
RDS	48	Poor responders to surfactant showed lower plasma C1q and C4 and higher plasma C3a and C5a 24hrs after birth (71)
RDS	35	There was no evidence of significant C activation in RDS (286)
ALI (allograft)	32/8	Pulmonary subendothelial C4d deposition was associated with HLA-Ab-induced ALI; elevated the plasma levels of C4d, Bb, iC3b, and C5b-9 after OKT3 administration in kidney and lung transplant recipients (287, 288)
Trauma-induced ARDS	208	Increased plasma C5b-9 was associated with ARDS development (94)
Trauma-induced ARDS	108	C activation was associated with ARDS development (5)
Trauma-induced ARDS	38	Increased plasma C3a was associated with ARDS development; C3a/C3 ratio discriminated ARDS and non-ARDS (53)
Trauma-induced ALI	54	Complement activation after traumatic injury, and complement activation is correlated with clinical outcomes in trauma patients (256)
Trauma/sepsis-induced ARDS	48	Plasma C3a was associated with ARDS development (289)
Trauma/sepsis-induced ARDS	26	Plasma C3a correlated with alveolar-capillary permeability (113)
Trauma/sepsis-induced ARDS	N/A	Plasma C1 inhibitor activity was significantly reduced in ARDS patients (290)
Sepsis-induced ARDS	87	Increased plasma C5b-9, C1rC1s-C1 inhibitor and C3bbP preceded ARDS development and resolution; plasma C5b-9 was more sensitive than C3a, C4a, C5a and CH50 (241, 291)
Sepsis-induced ARDS	48	Increased plasma C3a was useful for prognosis and diagnosis of sepsis and septic shock but not for ARDS (292)
Sepsis-induced ALI	40	Plasma C3a and C5a were increased in patients but did not predict the development of ALI (293)
H1N1-induced ARDS (H1N1)	97	Elevated baseline blood levels of MBL were associated with high mortality (NCT03641690)
COVID-19-induced ARDS	276	Complement consumption was associated with the severity of COVID-19 patients (165)
COVID-19-induced ARDS	100	Show an overexpression of C5a receptor in patients with ARDS secondary to COVID-19 compared to control patients (NCT04369820)
COVID-19-induced ARDS		Increased plasma levels of C3a, C3c and C5b-9 were related to disease severity in COVID-19 patients (48)
COVID-19-induced ALI	19	Elevated plasma levels of C3a, C3d/g, C4d, and C5b-9 were associated lung damage in COVID-19 patients (190)
COVID-19-induced ARDS/ALI		High expression levels of C3a, C3bc, C4bc, C5a and factor P were positively correlated with IL-8, CCL5, and the fatality rate in COVID-19 patients (294, 295)
COVID-19-induced ALI/ARDS		Significant deposits of C4d, C5b-9 and MASP-2, and colocalization of COVID-19 spike glycoproteins with C4d and C5b-9 in the interalveolar septa microvasculature in COVID-19 patients (57)
ECMO	2	Rapid activation of the complement alternative pathway by ECMO (296)

Ab, antibody; ALI, acute lung injury; ARDS, acute respiratory distress syndrome; BALF, bronchoalveolar lavage fluid; CCL5, C-C motif chemokine ligand 5; ECMO, extracorporeal membrane oxygenation; HLA, human leukocyte antigen; MASP-2, mannan-biding lectin serine protease 2; PFB, properdin factor B; RDS, respiratory distress syndrome; sCR1, soluble complement receptor 1; WBC, white blood cell; N/A, not available.

In clinical practice, there are only two classes of complement inhibitors available. Plasma-derived human C1 inhibitors [Cinryze (Shire), Berinert (SCL, Behring), and Cetor (Sanquin)] and recombinant C1 inhibitor (Ruconest, Parming) are already approved for hereditary angioedema (HAE) (153, 297). C5 inhibitor Eculizumab (Soliris, Alexion) is another FDA-approved drug for use in patients with paroxysmal nocturnal hematuria (PNH) (153, 297). Previous efforts in complement drug discovery were hampered by limited molecular insights, technical challenges, and safety concerns. After years of development, there are currently more than a dozen candidate drugs covering a wide range of the ComC being evaluated in clinical trials. They demonstrate a broad spectrum of therapeutic targets, in the use of molecular entities (small molecules, peptides, proteins, antibodies, and oligonucleotides), and expending indications and off-label use such as transplantation, SIRS, sepsis, inflammatory diseases, and chronic obstructive pulmonary disease (298).

With regard to targeting complement therapy in human studies of ALI/ARDS, there are currently seven interventional trials (**Table 7**). All studies have been designed to address the safety and efficacy of the complement inhibition by targeting C1q/MBL, C3, or C5 in COVID-19-induced ALI/ARDS (**Table 7**).

**Table 7:** Overview of clinical therapeutics for clinical trials in ARDS/ALI.

Target	Interventions	Indications	Primary endpoint	Participants	Status	References
C1/MBL	RLS-0071	COVID-19-induced severe ARDS	Frequency and severity of Adverse Events, including Serious Adverse Events, by treatment group and dose level, including the frequency of premature discontinuation of study intervention due to Adverse Events	42	Not yet recruiting	NCT04574869 (P1)
C3	AMY-101	COVID-19-induced severe ARDS	1) The proportion of patients who are alive, without evidence of ARDS; and 2) The proportion of patients assigned to each category, of a six-category ordinal scale.	144	Not yet recruiting	NCT04395456 (P1-P2)
C3	APL-9	COVID-19-induced severe ARDS	Cumulative incidence of treatment emergent serious adverse events and treatment-emergent adverse events	66	Recruiting	NCT04402060 (P2)
C5	Ravulizumab	COVID-19-induced severe ARDS/ALI/pneumonia	Survival (based on all-cause mortality) at Day 29	270	Active, not recruiting	NCT04369469 (P3)
C5	Zilucoplan^®^	COVID-19-induced severe ARDS	1) Mean change in oxygenation [Time Frame: at predose, day 6 and day 15 (or at discharge, whichever comes first)] defined by Pa02/FiO_2_ ratio while breathing room air, P(Aa)O_2_ gradient and a/A pO_2_ ratio; and 2) Median change in oxygenation [Time Frame: at predose, day 6 and day 15 (or at discharge, whichever comes first)] defined by PaO_2_/FiO_2_ ratio while breathing room air, P(Aa)O_2_ gradient and a/A pO_2_ ratio	81	Completed	NCT04382755 (P2)
C5	SOLIRIS^®^	COVID-19-induced severe ARDS/ALI/pneumonia		N/A	No longer available	NCT04355494 (P2)
C5	SOLIRIS^®^	COVID-19	Mortality	N/A	Available	NCT04288713

ALI, acute lung injury; ARDS, acute respiratory distress syndrome; MBL, mannan-biding lectin; N/A, not available.

## 6 Conclusions and future directions

Although the many roles of complement-mediated pathomechanisms and various promising preclinical results of targeting complement therapies in ALI/ARDS have long been recognized, clinical trials controlling complement activation in ALI/ARDS remain rare. Despite this improved understanding, no pharmacological therapeutics targeting ComC have been approved for ALI/ARDS patients. Increasingly, ARDS have been recognized as a heterogeneous syndrome characterized by subphenotypes with distinct etiologies, pathophysiological/radiographic features, and clinical outcomes. These subphenotypes have potentially distinct ComC activation profiles (endotypes) and different responses to targeting ComC therapeutics (theratypes). Thus, there is increasing interest in better defining complement endotypes and theratypes within individual ALI/ARDS patients in order to facilitate targeting ComC personalized/precision medicine. However, several challenges still exist including (1) the lack of point-of-care diagnostic devices to timely measure the targeted complement factors or activation products and real-time monitor the dynamic change of complement activation and immune status caused by ALI/ARDS and/or post-ARDS therapeutic approaches (blood transfusion, ECMO, damage control surgery, ventilation, volume resuscitation, etc.) (214, 299); and (2) the lack of a simple prognostic/diagnostic test to identify endotypes/theratypes of ARDS because ARDS is an interpatient heterogeneous syndrome that can have a differential ComC response to the same etiology or the heterogeneous etiologies of ALI/ARDS and a different response to targeting complement therapies (300). However, the future looks much brighter due to the increasing availability of various highly effective, specific complement therapeutics developed over the last two decades targeting different components of the ComC, with different half-lives, different routes of administration, lower cost, and lower infection risk in the context of inflammation, trauma, hemorrhage, and sepsis (297, 301). Therefore, future work should focus on furthering our mechanistic understanding of ARDS endotypes/theratypes, integrating preexisting complement functions on immunome, coagulome, metabolome, microbiome, genome, epigenome, proteome, neuroendocrinome and lipidome, establishing pulmonary drug delivery, identifying specific biomarkers of ARDS and immune status, developing multiplexed point-of-care testing, and utilizing multi-omics approach to define endotypes/theratypes aimed at applying targeting complement therapies to those ARDS patients who are most likely to respond to them.

## Author contributions

Conceptualization, YL and LC. Writing original draft, ZY. Writing/review/editing, YL, ZY, LC, SN, and TC. All authors contributed to the article and approved the submitted version.

## Glossary

**Table:**

Biomarker	measurable substance linking disease endotype with the phenotype
Coagulome	an overview of the equilibrium between the coagulation and fibrinolysis
Complementome	an overview of the extracellular and intracellular ComC
Complosome	an intracellularly active complement system
Connectome	the comprehensive description of the signaling pathway connectivity
DAMPome	an overview of the extracellular and intracellular DAMPs
Endotype	compilation of functional/pathobiological mechanisms explaining disease expression in groups of patients
Immunome	an overview of the complete whole of immune system
Metabolome	a complete set of the metabolism
Microbiome	a complete set of the community of microorganisms
PAMPome	an overview of the extracellular and intracellular DAMPs
Phenotype	observable characteristics/traits attempting to individualize disease expression in groups of patients
Theratype	prediction of treatment response based on the disease endotype
